# Placement of bronchial occluder outside the tracheal tube in a patient combined with airway compression undergoing mediastinal tumors resection: a case report

**DOI:** 10.1186/s12871-024-02480-2

**Published:** 2024-03-12

**Authors:** Yihu Zhou, Yueyi Jiang, Yuyan Ding, Lianbing Gu, Jing Tan

**Affiliations:** Department of Anesthesiology, The Affiliated Cancer Hospital of Nanjing Medical University, Nanjing, 21009 People’s Republic of China

**Keywords:** Mediastinal tumors, Tracheal compression, One-lung ventilation, Bronchial occluder

## Abstract

**Background:**

Mediastinal tumors pose a challenging respiratory and circulatory management during anesthesia procedures, there is a risk of circulatory collapse or complete airway obstruction, which in severe cases can lead to cardiac arrest. We reported a case of anesthetic management using a bronchial blocker placed outside the tracheal tube. In this case report, the patient’s trachea was so severely compressed that the airway was extremely narrow, only 4 mm at its narrowest point. By reporting the anesthetic management of this patient, we intend to provide an unusual approach for airway management.

**Case presentation:**

A 52-year-old male patient was admitted to the hospital due to cough and expectoration for one year. Additionally, the patient experienced chest tightness and asthma after physical activity. The enhanced computed tomography revealed there existed an irregular soft tissue mass in the right upper mediastinum, which significantly compressed the trachea and esophagus. The results of the mediastinal puncture pathology showed the presence of mesenchymal tumors. According to the results above, the patient was diagnosed with a mediastinal tumor and scheduled to undergo tumor resection under general anesthesia. We used a bronchial occluder outside the tracheal tube for general anesthesia. After surgery, the patient received thorough treatment and was subsequently discharged from the hospital.

**Conclusion:**

In patients with severe airway compression from a mediastinal tumor airway compression, positioning a bronchial occluder externally to the tracheal tube is an effective method of airway management. However, we still need more clinical practice to help the process become more standardized.

## Background

Mediastinal tumors may cause compression of essential blood vessels, the trachea or bronchus, and other vital organs, which can significantly impact the cardiovascular and respiratory systems. It requires targeted treatment measures based on a precise diagnosis, most of which require surgical resection [1, 2]. The risk of general anesthesia is extremely high in patients with mediastinal tumors when they are accompanied by obstruction of the large airways. After inducing and maintaining anesthesia, and following postoperative extubation, there is a risk of circulatory collapse or complete airway obstruction, which in severe cases can lead to cardiac arrest [3–6]. It is crucial to ensure proper perioperative management and thorough care to prevent the worsening of compression syndrome. In this report, one patient who suffered airway compression with a mediastinal tumor was successfully underwent mediastinal tumor resection with the insertion of a new bronchial occluder device outside the tracheal tube for general anesthesia, we discuss the anesthesia management of this patient, particularly the airway management.

## Case presentation

The 52-year-old male patient was admitted to the hospital due to complaints of chest tightness and shortness of breath. Subsequent diagnosis revealed the presence of a mediastinal tumor. The patient weighed 76 kg, was 170 cm tall, had no history of obstructive sleep apnea, previous anesthesia, or drug use. The airway assessment revealed regular characteristics, with a modified Mallampati classification of II. Transthoracic echocardiography indicated no abnormalities in cardiac structure and function. The electron bronchoscopy report revealed compression of the trachea by a mediastinal mass, resulting in the narrowing of the airway.

From the incisors to the trachea’s narrowest point was measured 22 cm, roughly four cartilaginous rings away beyond the glottis. The narrowest point measured at 4 mm, permitting passage only for the fibrobronchoscope (4.9 mm).

Intraoperatively, electrocardiogram, pulse oxygen saturation, invasive blood pressure (BP), bispectral index (BIS), nasopharyngeal temperature, and urine output were monitored. We performed a right radial artery puncture and cannulation to monitor arterial blood pressure and blood gas, we also established femoral vein access. Concurrently we prepared rescue medications including atropine, norepinephrine, epinephrine, and sugammadex sodium. A flexible guidewire(1 mm) was inserted into the bronchial occluder(3 mm), and its end was connected to the standby anesthesia machine. This modification allowed us to convert a conventional bronchial occluder into one that could be ventilated and adjusted in direction.

**Fig. 1:**
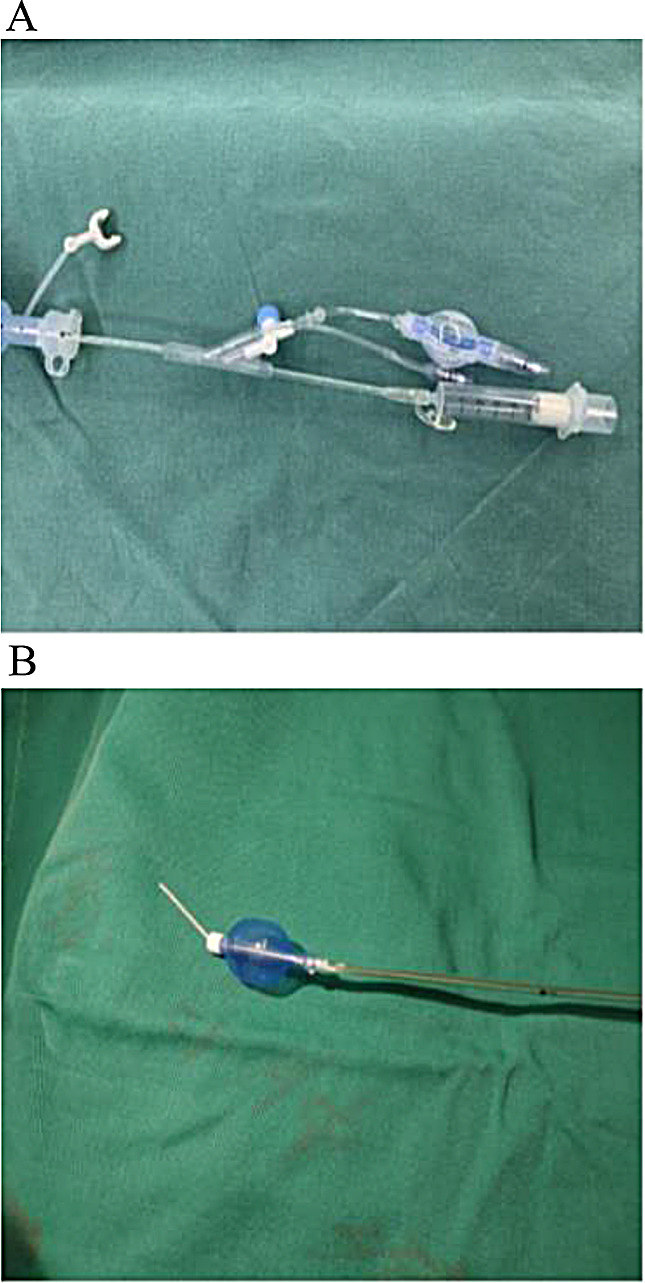
**A** Ventilation connection at the end of the bronchial blocker; **B** Insult an elastic bougie to adjust the direction of the bronchial occluder

We opted for tracheal intubation using a bronchial occluder placed outside the tracheal tube to facilitate airway management. The patient’s position was tilted 30 degrees to the right, and oxygen was delivered via a face mask. Anesthesia was induced intravenously with dexamethasone (5 mg), oxycodone (3 mg), 1% propofol (20 mg·kg^-1^), sufentanil (0.4 µg·kg^-1^), and rocuronium (0.6 mg·kg^-1^). Once the patient’s muscles were relaxed enough for intubation, a visual laryngoscope was used to expose the glottis, and a self-made plastic bronchial occluder (Fig. 1. A, B) connected to the standby anesthesia machine was inserted, connected to the standby anesthesia machine was inserted. The bronchial occluder was gradually adjusted, guided by a fibrobronchoscope, to navigate through the tracheal stenosis area.

We rotated and inserted the bronchial occluder into the right bronchus, inflated the cuff of the bronchial occluder, and confirmed the proper position using a stethoscope and fibrobronchoscope. An ID 6.0 mm tracheal tube was inserted under the guidance of a video laryngoscope and fibrobronchoscope, positioned 3 cm away from the tracheal carina. The position of the bronchial blocker cuff was reconfirmed. The parameters applied during two-lung ventilation were: tidal volume of 8 ml·kg^− 1^ (predicted body weight); PEEP 5 cmH_2_O; FiO_2_ 0.5–0.8; inspiratory to expiratory ratio 1:2; and respiratory rate adjusted to maintain ETCO_2_ between 35 and 45 mmHg. During one-lung ventilation, the parameters were adjusted to a tidal volume of 6 ml·kg^−^¹ (predicted body weight); PEEP 5 cmH_2_O; FiO_2_ 0.4–0.5; inspiratory to expiratory ratio 1:2; and respiratory rate adjusted to maintain ETCO_2_ between 35 and 45 mmHg. Anaesthesia was maintained through infusions of propofol(0.05 mg·kg^− 1^·min^− 1^), remifentanil(0.2 µg·kg^− 1^·min^− 1^), rocuronium(0.01 mg·kg^− 1^·min^− 1^) and dexmedetomidine(16 µg·h^− 1^). Ephedrine or phenylephrine was administered when systolic blood pressure decreased below 90 mmHg. During the surgery, we used a forced-air warming device to maintain the nasopharyngeal temperature above 36ºC.

The operation lasted for 205 min. The mediastinal mass, measuring 10 cm*6 cm *6 cm, was successfully resected, and the patient’s circulation was stable during the operation. Upon completion of the surgical procedure, the bronchial blocker was removed, and the recruitment maneuver was subsequently performed(The anesthesia machine was set to CPAP mode, adjusting PEEP to 30 cmH_2_O, maintained for 30 s). Meanwhile, a fibrobronchoscope was used to ensure the absence of tracheal malacia and collapse. Pain control in the postoperative period was managed through Patient-Controlled Intravenous Analgesia (PCIA) with a drug formula consisting of sufentanil (150 ug) and tropisetron (8 mg) diluted in physiological saline to 100 ml. The continuous infusion rate was set at 1.5 ml·h^-1^for 48 h.

The patient was successfully extubated in the ICU on the second postoperative day and discharged from the hospital on the fifth postoperative day. A follow-up conducted three months later via telephone revealed no adverse events. Written informed consent was obtained from the patient to publish this case report.

## Discussion and conclusions

Patients with symptoms indicative of tracheal compression due to a mediastinal tumor may exhibit varying degrees of ventilatory dysfunction. The goal of surgery is to alleviate airway compression and improve ventilation, necessitating thorough preoperative assessment, the formulation of an individualized airway management plan, and vigilant intraoperative anesthetic monitoring. Before administering anesthesia, it is critical to obtain a comprehensive medical history, assess symptoms and signs, and review the surgical plan to evaluate perioperative risk factors. Anesthesiologists are seeing their role expand due to their unique and intensive training on the specific demands of surgical procedures [7]. Effective communication between surgical and anesthetic teams is crucial during the pre-anesthetic assessment to ensure the implementation of an appropriate anesthetic protocol. Special attention should be paid to patients who have demonstrated positional respiratory distress and ensure that the patient is in an appropriate position to minimize the risk of airway obstruction.

Patients with mediastinal masses are at risk of central airway occlusion during general anesthesia [8]. To reduce the risk, previous studies are recommended to maintain spontaneous ventilation and avoid neuromuscular blockade [9, 10]. However, recent studies have found no worsening of symptoms of airway compression when anesthesia is induced with staged positive-pressure ventilation and adequate muscle relaxation in adult patients with large mediastinal masses [11].

Mediastinal tumor resection usually requires one-lung ventilation to ensure a clear surgical field.

Currently, the double-lumen tube is the preferred approach for one-lung ventilation. However, intubation in patients with malignant airway stenosis is often challenging, and difficulty with the airway and tracheal stenosis are both contraindications to the use of a double-lumen trachea, either of which may prevent proper positioning of the catheter [12].

In the present case, a CT scan revealed that the patient’s trachea measured only 4 mm at its narrowest point, and was classified as Myer-Cotton grade III [13, 14], rendering the passage of a 32 F double-lumen bronchial tube unfeasible. a single-lumen tracheal tube may be more appropriate for its thinner diameter. To meet the requirements of one-lung ventilation, a bronchial occluder was initially placed, followed by the intubation of an ID 6.0 mm tracheal tube. This approach aimed to increase the ventilatory area and ensure sufficient intraoperative ventilation. The ventilatory area of an ID 6.0 mm tracheal tube is 28.3 mm^2^. However, with the placement of a blocker(3 mm) within the tube, the ventilatory area decreases by 25%. Subsequently, the ventilated area is reduced to 21.2 mm², equivalent to the ventilated area of an ID 5.0 mm single-lumen tracheal tube. Therefore, the use of bronchial occluder placement outside the tracheal tube for airway management can mitigate airway pressure, reduce the risk of lung injury, and facilitate the use of fibrobronchoscope for position confirmation [15, 16].

The combined use of the laryngeal mask and bronchial blocker presents a potential strategy for airway management in this patient. However, it warrants consideration that the laryngeal mask may be susceptible to dislocation in response to changes in body position [17].

Moreover, intervention itself has the potential to exacerbate tracheal stenosis, thereby posing a substantial challenge to intraoperative airway control. The implementation of cardiopulmonary diversion or extracorporeal membrane oxygenation (ECMO) stands as a plausible alternative. Nonetheless, a judicious evaluation of the associated complications and financial considerations is imperative prior to committing to ECMO as a viable course of action [18].

After comprehensive consideration, we opted for the approach of positioning a bronchial occluder externally to the tracheal tube to facilitate one-lung ventilation in this case. Meanwhile, we also used a custom-designed external bronchial occluder ventilation device connected to the anesthesia machine. This ventilation device addresses concerns related to decreased oxygen saturation during intubation caused by positioning and prolonged endotracheal tube intubation [19]. Meanwhile, studies have shown that intraoperative low-flow oxygen administration in the non-ventilated lung can alleviate non-ventilated lung injury [20]. In addition to this, the custom-designed device allows for orientation adjustment during the placement of the bronchial occluder. During the procedure, it took approximately 2 min to place the blocker. The blocker was repositioned only once when the patient changed to the lateral position; Additionally, throughout the entire surgical procedure, we used a fiberoptic bronchoscope to inspect and confirmed that the position of the bronchial blocker remained unchanged. During the entire surgical procedure, the patient did not experience any state of desaturation.

The selection of the type of bronchial blocker is also important, the bronchial occluder used in this case report is a coopdech blocker used for placing outside the tracheal tube. Due to its similar design to the coopdech blocker, it may also be feasible to attempt the use of Arndt blocker in such cases. In contrast, the Univent blocker may not be suitable for such cases due to its larger diameter and smaller lumen ventilation area, as well as its harder material, which can easily damage the airway [21]. Cohen blocker is not recommended for use as its blocked tube does not have a lumen and cannot assist in non-ventilated lung collapse and CPAP procedures. In addition, the Tritube is a narrow bore cuffed endotracheal tube with inner and outer diameters of 2.4 and 4.4 mm. It provides complete ventilation using a mere 2 to 3 mm catheter. The ventilation lumen is attached to the Ventinova. This device may also be useful in such emergency airway case settings [22].

Previous research reports on patients with mediastinal tumors leading to airway compression have provided us with many hints in the event of hypoxemia in patients. For unexplained hypoxemia during single lung ventilation, echocardiography may be used for evaluation [23]. High-frequency jet ventilation and title of positive end-expiratory pressure to the dependent lung can be used on the non-ventilated side to alleviate hypoxemia [24].In emergencies where mechanical ventilation cannot meet the oxygen supply during surgery, VV-ECMO technology can be used [25]. In this case, we prepared High-frequency jet ventilation during the surgery, but the patient’s oxygenation remained stable after using non-ventilated lung CPAP, so the above protocol was not used.

In addition to the appropriate choice of airway management, comprehensive intraoperative and postoperative management is essential. During surgery, vigilant monitoring of ECG, non-invasive blood pressure, SpO_2_, and P_ET_CO_2_ is crucial, along with invasive blood pressure measurements and arterial blood gas analysis to mitigate the risk of hypoxia and CO_2_ retention. Isolating the mediastinum tumor during surgery may result in compression of vital structures such as the heart, aorta, and superior vena cava, potentially causing circulatory fluctuations. Additionally, vascular compression, stretching, or blood loss may lead to hemodynamic instability. Therefore, preoperative placement of a central venous catheter in the femoral vein as a rehydration channel is deemed necessary. Intraoperatively, vasoactive drugs are required to maintain blood pressure if necessary. Considering the invasive and prolonged nature of the procedure, safeguarding body temperature and continuous temperature monitoring are paramount aspects of perioperative management [26].

After surgery, the patients need to make a thorough assessment and preparation before they can be extubated. The use of a peripheral nerve stimulator to ensure a train-of-four ratio of 0.9 and use the Sugammadex if necessary; Maintain stable hemodynamics to avoid hypertension or hypotension after extubation; Prevent hypothermia causing delayed awakening; Adequate analgesia should be provided to avoid agitation during awakening [27]. At the time of extubation, a thinner guide tube can be placed in the tracheal tube to allow for reintubation. If necessary, take the patient to the intensive care unit for further observation before extubation.

In presenting our anesthetic strategy for a patient with pronounced airway compression from a mediastinal tumor, we underscore the novel approach of employing an external bronchial occluder alongside the tracheal tube for optimized airway management. Although this method demonstrates potential, its comprehensive integration into diverse clinical settings remains an area for further exploration. Addressing its practical limitations and refining specific protocols will be instrumental in elevating the method’s effectiveness across a broader range of clinicals.

## Data Availability

This manuscript does not report data generation or analysis.

## Abbreviations

CT: Computed Tomography
ABP: Arterial Blood Pressure
ASA: American Society of Anesthesiologists
PCIA: Patient-Controlled-Intravenous-Analgesia
ECMO: Extracorporeal Membrane Oxygenation
